# Incidence of ocular pathology following bariatric surgery for with morbid obesity across a large United States National Database

**DOI:** 10.1038/s41433-024-03088-z

**Published:** 2024-04-27

**Authors:** Matthew W. Russell, Madhukar Kumar, Ang Li, Rishi P. Singh, Katherine E. Talcott

**Affiliations:** 1 Cleveland Clinic Lerner College of Medicine of Case Western Reserve University, Cleveland, OH USA; 2 Center for Ophthalmic Bioinformatics, Cole Eye Institute, Cleveland Clinic, Cleveland, OH USA; 3 Case Western Reserve University School of Medicine, Cleveland, OH USA; 4 Cleveland Clinic Cole Eye Institute, Cleveland, OH USA; 5 Martin North Hospital, Cleveland Clinic, FL USA

**Keywords:** Metabolic disorders, Retinal diseases

## Abstract

**Background/Objectives:**

Bariatric surgery, as indicated for treatment of morbid obesity, has been studied in association with short term effects on ocular pathology. However, effects of surgery on postoperative disease incidence is largely unknown.

**Subjects/Methods:**

In this retrospective cohort study, the TriNetX United States Collaborative Network national database, was queried for patients with an ICD-10 code for morbid obesity and a procedural code for bariatric surgery. Patients were propensity score matched across baseline demographics at the time of surgery and compared to those presenting with an ICD10 code for morbid obesity with no records of a procedural code for bariatric surgery, identifying 42,408 patients per cohort. New diagnoses or procedural codes found after the surgical index date for diabetic retinopathy, age-related macular degeneration, glaucoma, low vision, and blindness along with pertinent treatment metrics were monitored.

**Results:**

Bariatric surgery was found to be associated with reduced future risk of diabetic retinopathy (RR: 0.283; 95% CI: 0.252–0.319), macular edema (RR: 0.224; 95% CI: 0.170–0.297), vitreous hemorrhage (RR: 0.459; 95% CI: 0.323–0.653), ocular hypertension (RR: 0.387; 95% CI: 0.387–0.487), glaucoma (RR: 0.360; 95% CI: 0.326–0.399), use of ocular pressure lowering medications (RR: 0.565; 95% CI: 0.496–0.644), age-related macular degeneration (RR: 0.628; 95% CI: 0.447–0.882), cataract surgery (RR: 0.524; 95% CI: 0.448–0.612), and low vision and blindness (RR: 0.328; 95% CI: 0.294–0.365) compared to patients not surgically managed.

**Conclusions:**

The present analysis comprising a large US cohort of patients suggests that bariatric surgery is associated with a decreased risk of future ocular morbidity and mortality.

## Introduction

As obesity co-exists with and exacerbates systemic health conditions, obesity has been associated with increased risk of all-cause mortality [1]. While diet and weight loss efforts are often first line in mitigation of obesity and restoration of normal weight, intensive surgical interventions such as bariatric surgery is an increasingly utilized option for patients who present with a body mass index (BMI) above 40 and are refractive to conservative medical management [2]. Since the advent of bariatric surgery, the efficacy of this procedure on weight loss and systemic health has been profound. Bariatric surgery has been shown in large clinical trials to reduce mean BMI, along with risk factors for cardiovascular disease and metabolic syndrome [3, 4].

Obesity has been implicated as a driver of various ocular disease states, increasing risk for diabetic retinopathy, glaucoma, and age-related macular degeneration [5]. Due to these associations, increasing attention has been focused on effects of bariatric surgery and mitigation of ocular pathology. A two-year analysis of the STAMPEDE trial, a prospective randomized trial examining bariatric surgery compared to intensive medical management for treatment of morbid obesity, showed patients treated with bariatric surgery to experience no worsening or benefit with respect to diabetic retinopathy (DR) outcomes [6]. An subsequent 5-year analysis further found no differences in DR worsening or improving in patients undergoing bariatric surgery compared to medically managed patients [7]. However, a recent systematic review pooling 876 patients undergoing bariatric surgery showed patients who underwent surgery were at decreased odds of DR progression than those who were obese and did not undergo surgery [8].

With respect to other ocular pathologies, small cohorts of patients undergoing bariatric surgery have also shown decreases in intraocular pressure (IOP) short term post-surgery [9, 10]. However long-term effects examining development of glaucoma have yet to be examined. Zhang et al. explored a pooled analysis of studies examining age related macular degeneration (AMD) and body mass index BMI, finding every 1 kg/m^2^ increase in BMI incurred a 2% increased risk of carrying a diagnosis of AMD [11]. While these results seem promising, no studies to date have examined odds of developing AMD after intervention with bariatric surgery. Obesity has been found to incur increased risk of cataract formation [12, 13]. Recently, Burkard et al. examined patients undergoing bariatric surgery, finding them to be at significantly decreased risk of cataract formation [14]. However, this design did not stratify by types of cataract, and need for future cataract surgery, leaving areas for continued investigation.

Although the associations between bariatric surgery and development of ocular disease are mixed, studies to date largely reveal protective trends. Furthermore, these associations have yet to be explored in the context of large U.S. cohort. The advent of TriNetx, a large U.S. collaborative national database, allows such studies to be performed. Herein, this study aims to characterize the effect of bariatric surgery on post-operative prevalence of diabetic retinopathy, glaucoma, age-related macular degeneration, blindness, and associated disease specific worsening events using the TriNetX U.S. Collaborative network.

## Materials/subjects and methods

This study was performed utilizing the TriNetX U.S. Collaborative Network: a large database containing aggregated, standardized, and de-identified medical records data that utilizes International Classification of Diseases (ICD) codes. The TriNetX U.S. Collaborative Network provides access to electronic medical records information on approximately 86 million patients from 54 contributing healthcare organizations. TriNetX, LLC is a Health Insurance Portability and Accountability Act (HIPAA) compliant, certified to the ISO 27001:2013 standard, and maintains an Information and Security Management System to ensure protection of data.

Using the TriNetX database, patients were queried for presence of a procedural code for bariatric surgery (43,774) and international classification of disease-10 (ICD-10) code for morbid obesity (E66.01), identifying 42,408 patients. A control cohort of patients with a diagnosis of morbid obesity (E66.01) and no procedural code for bariatric surgery (43774) was also queried, identifying 972,234 patients. To achieve balance amongst cohorts, propensity score matching was performed using the TriNetX analytic feature (greedy matching, calliper of 0.25 SD). The following criteria were matched upon: age, gender, race, ethnicity, BMI, hypertension (HTN), and hemoglobin A1c.

Once cohorts were obtained, development of pertinent ICD-10 codes were queried for after the date of bariatric surgery for surgical patients or after a diagnosis of morbid obesity in control cohorts as seen in Supplementary Table 1. For surgical patients, the analysis window was any date after bariatric surgery. For non-surgical patients, the analysis window was any time after the diagnosis of morbid obesity. To ensure only incident cases were included in the analysis, patients with an ICD pathology, procedure, or medication code of interest before the surgical or matching date were excluded from the analysis. Risk ratios were calculated to determine the impact of a bariatric surgery on the risk of new ocular diagnoses. Risk ratios were reported, along with confidence intervals.

## Results

### Demographics

In the TriNetX national database, a total of 42,408 patients were identified to carry a diagnosis of morbid obesity and underwent bariatric surgery. A total of 831,791 patients were identified to carry a diagnosis of morbid obesity lacking a procedural code for bariatric surgery. Propensity score matching was used as stated above. Before matching, patients in the bariatric cohort were significantly younger, included a greater percentage of female patients, and were more obese than those who were not treated surgically. After matching, each cohort contained 42,408 patients and patient cohorts were more similar with respect to age, gender, race, BMI, HbA1c, and HTN status although differences were still present (Table 1).

**Table 1:** Demographic information of Bariatric surgery and Obese cohorts.

Variable	Bariatric surgery [SD] or (%)	Obese [95% SD] or (%)	*p* value
	(*N* = 42,408)	(*N* = 42,408)	
Age (years)	43.2 +/− 12.4	43.6 +/− 13.9	**<0.001**
Gender			
Female	34,273 (82.3%)	34,323 (80.9%)	0.662
Male	8131 (19.2%)	8073 (19.0%)	0.612
Race			
White	27,307 (64.4%)	27,961 (65.9%)	**<0.001**
Black	11,237 (26.5%)	10,639 (25.1%)	**<0.001**
Ethnicity			
Hispanic	3772 (8.9%)	3300 (7.8%)	**<0.001**
BMI			
BMI	42.9 +/− 5.4	40.8 +/− 5.5	**<0.001**
Labs			
HbA1c	6.03 ± 1.24	6.2 ± 1.6	**<0.001**
LDL cholesterol	106 ± 32.8	106 ± 33.1	0.076
Systemic diagnoses			
Essential HTN	23,378 (55.1%)	23,542 (55.5%)	0.257
Type 1 DM	1230 (2.9%)	1126 (2.7%)	**0.029**
Type II DM	12,055 (28.4%)	11,674 (27.5%)	**0.003**
Long term use of insulin	2176 (5.1%)	2074 (4.8%)	0.108
Hypercholesterolemia	5670 (13.4%)	5504 (12.9%)	0.092
CAD	2623 (6.2%)	2403 (5.1%)	**0.005**
OSA	21,005 (49.5%)	20,778 (48.9%)	0.119
Ocular diagnoses			
Type I DM with DR	90 (0.2%)	69 (0.2%)	0.095
Type II DM with DR	644 (1.5%)	450 (1.1%)	**<0.001**
non exudative AMD	30 (0.1%)	30 (0.1%)	1
exudative AMD	10 (0.1%)	15 (0.1%)	0.317
Glaucoma suspect	661 (1.4%)	467 (0.9%)	**<0.001**
OHTN	195 (0.5%)	153 (0.4%)	**0.024**
Glaucoma	926 (2.2%)	738 (1.7%)	**<0.001**
Blindness and low vision	651 (1.5%)	537 (1.3%)	**<0.001**

Statistically significant values are in bold.

*SD* standard deviation, *BMI* body mass index, *HTN* Hypertension, *HbA1c* hemoglobin A1c, *LDL* low density lipoprotein, *DM* diabetes mellitus, *CAD* coronary artery disease, *OSA* obstructive sleep apnea, *DR* diabetic retinopathy, *AMD* age related macular degeneration, *OHTN* ocular hypertension.

### Ocular outcomes

Of the 42,408 patients who underwent bariatric surgery, 347 of them were given a new diagnosis of any type of diabetic retinopathy after their surgical date. Of the patients who did not undergo surgery, 1232 of them experienced a new diagnosis of diabetic retinopathy in the future as seen in Table 2. Patients who underwent surgery had significantly reduced risk of developing new diabetic retinopathy compared to those who were not (RR: 0.283; 95% CI: 0.252–0.319). When stratified by category of DR, patients who were surgically managed were significantly less likely to experience nonprolifereative DR (RR: 0.324; 95% CI: 0.274–0.382) or proliferative DR (RR: 0.453; 95% CI: 0.339–0.604) compared to those who did not undergo surgery (Table 2). Patients who underwent bariatric surgery were also significantly less likely to experience complications of diabetic retinopathy worsening such as vitreous hemorrhage, intravitreal injection, pars plana vitrectomy, pan retinal photocoagulation, and tractional retinal detachment (*p* < 0.05 for all, Table 2).

**Table 2:** Risk of post operative retinal pathology and complications after surgery.

Cohorts	Patients in cohort	Patients with outcome	Risk ratio (CI)	*P* value
All forms of diabetic retinopathy				
Bariatric surgery	41,661	347	0.283 (0.252, 0.319)	**<0.001**
Obese	41,880	1232		
Nonproliferative diabetic retinopathy				
Bariatric surgery	42,156	183	0.324 (0.274, 0.382)	**<0.001**
Obese	42,221	566		
Mild nonproliferative diabetic retinopathy				
Bariatric surgery	42,197	160	0.334 (0.279, 0.399)	**<0.001**
Obese	42,247	480		
Moderate nonproliferative diabetic retinopathy				
Bariatric surgery	42,341	54	0.342 (0.251, 0.466)	**<0.001**
Obese	42,365	158		
Severe nonproliferative diabetic retinopathy				
Bariatric surgery	42,376	25	0.373 (0.236, 0.591)	**<0.001**
Obese	42,383	67		
Proliferative diabetic retinopathy				
Bariatric surgery	42,295	67	0.453 (0.339, 0.604)	**<0.001**
Obese	42,316	148		
Diabetic retinopathy with macular edema				
Bariatric surgery	42,046	60	0.224 (0.17, 0.297)	**<0.001**
Obese	42,150	268		
Vitreous Hemorrhage				
Bariatric surgery	42,351	45	0.459 (0.323, 0.653)	**<0.001**
Obese	42,348	98		
Intravitreal injection				
Bariatric surgery	42,339	83	0.522 (0.4, 0.68)	**<0.001**
Obese	42,334	159		
Pars Plana Vitrectomy				
Bariatric surgery	42,381	23	0.46 (0.281, 0.754)	**0.001**
Obese	42,394	50		
Pan Retinal Photocoagulation				
Bariatric surgery	42,367	21	0.467 (0.278, 0.783)	**0.003**
Obese	42,378	45		
Tractional Retinal Detachment				
Bariatric surgery	42,391	10	0.476 (0.224, 1.011)	**0.048**
Obese	42,388	21		
All forms of AMD				
Bariatric surgery	42,370	54	0.628 (0.447, 0.882)	**0.006**
Obese	42,366	86		
Non exudative AMD				
Bariatric surgery	42,378	47	0.671 (0.464, 0.971)	**0.033**
Obese	42,378	70		
Exudative AMD				
Bariatric surgery	42,399	15	0.5 (0.269, 0.929)	**0.025**
Obese	42,393	30		
Retinal vascular occlusion				
Bariatric surgery	42,362	53	0.746 (0.523, 1.064)	0.105
Obese	42,331	71		
Retinal vein occlusion				
Bariatric surgery	42,386	32	1 (0.613, 1.632)	**0.999**
Obese	42,376	32		
Retinal artery occlusion				
Bariatric surgery	42,396	16	0.762 (0.398, 1.46)	**0.411**
Obese	42,386	21		

Statistically significant values are in bold.

*AMD* Age related macular degeneration.

Patients who underwent surgery were significantly less likely to be newly diagnosed with all types of AMD (RR: 0.628; 95% CI: 0.447–0.882) than those who did not have bariatric surgery. When stratified by type of AMD, patients in the bariatric cohort were still significantly less likely to experience nonexudative AMD (RR: 0.671; 95% CI: 0.464–0.971) and exudative AMD (RR: 0.500; 95% CI: 0.269–0.929) (Table 2).

While less cases of retinal vascular occlusion were observed in the surgically treated cohort, these differences were not found to be significant (RR: 0.746; 95% CI: 0.523–1.064). When stratifying by retinal vein or artery occlusion, no associations were uncovered (*p* > 0.05, Table 2).

Patients who underwent surgery were significantly less likely to present with new onset ocular hypertension (RR: 0.387; 95% CI: 0.308–0.487) or be diagnosed as a glaucoma suspect (RR: 0.406; 95% CI: 0.358–0.461) than those did not undergo surgery. Regarding all glaucoma associated diagnoses, patients undergoing surgery were significantly less likely to develop associated disease states (RR: 0.365; 95% CI: 0.330–0.403) as seen in Table 3. Concerning clinical management, patients undergoing surgery were significantly less likely to require pressure lowering medications (RR: 0.565; 95% CI: 0.496–0.644), or undergo future glaucomatous procedures or surgeries (RR: 0.461; 95% CI: 0.340–0.626). Finally, surgical patients were less likely to be diagnosed with new OSA (RR: 0.267; 95% CI: 0.250–0.285).

**Table 3:** Risk of post-operative glaucomatous diseases and complications in cohorts.

Cohorts	Patiens in cohort	Patients with outcome	Risk ratio (CI)	*P* value
Ocular hypertension				
Bariatric surgery	42,211	101	0.387 (0.308, 0.487)	**<0.001**
Obese	42,252	261		
Glaucoma suspect				
Bariatric surgery	41,733	335	0.406 (0.358, 0.461)	**<0.001**
Obese	41,926	829		
Ocular hypertension + Glaucoma suspects				
Bariatric surgery	41,733	335	0.406 (0.358, 0.461)	**<0.001**
Obese	41,926	829		
Glaucoma				
Bariatric surgery	41,445	501	0.36 (0.326, 0.399)	**<0.001**
Obese	41,626	1396		
Primary Open Angle Glaucoma				
Bariatric surgery	42,292	67	0.466 (0.349, 0.622)	**<0.001**
Obese	42,328	144		
Glaucomatous procedures and surgeries				
Bariatric surgery	42,300	60	0.461 (0.34, 0.626)	**<0.001**
Obese	42,292	130		
Use of pressure lowering eye drops				
Bariatric surgery	41,848	351	0.565 (0.496, 0.644)	**<0.001**
Obese	41,918	622		
Obstructive sleep apnea				
Bariatric surgery	19,184	991	0.267 (0.25, 0.285)	**<0.001**
Obese	20,703	4008		
Glaucomatous optic atrophy				
Bariatric surgery	42,363	33	0.611 (0.396, 0.942)	**0.024**
Obese	42,371	54		
Glaucoma Diagnoses (Glaucoma + Primary Open Angle Glaucoma + Glaucomatous Optic Atrophy)				
Bariatric surgery	41,429	513	0.365 (0.33, 0.403)	**<0.001**
Obese	41,605	1413		
Mild glaucoma				
Bariatric surgery	42,355	65	0.747 (0.542, 1.03)	0.075
Obese	42,373	87		
Moderate glaucoma				
Bariatric surgery	42,368	31	0.62 (0.396, 0.97)	**0.035**
Obese	42,376	50		
Severe glaucoma				
Bariatric surgery	42,381	19	0.704 (0.391, 1.265)	0.238
Obese	42,380	27		

Patients treated surgically significantly less likely to receive new diagnoses of cataract formation including age-related cataract, nuclear sclerotic cataract, cortical cataract, posterior subcapsular cataract, and diabetic cataract (*p* < 0.001 for all, Table 4). Surgically managed patients were also at significantly decreased risk of future cataract surgery than those not managed with surgery (RR: 0.524; 95% CI: 0.448–0.612).

**Table 4:** Risk of post-operative cataract formation and cataract surgery.

Cohorts	Patients in cohort	Patients with outcome	Risk ratio (CI)	P value
Age related cataract				
Bariatric surgery	41,378	928	0.479 (0.444, 0.518)	**<0.001**
Obese	41,124	1925		
Nuclear sclerotic cataract				
Bariatric surgery	41,565	758	0.483 (0.444, 0.527)	**<0.001**
Obese	41,400	1562		
Cortical cataract				
Bariatric surgery	42,296	113	0.451 (0.362, 0.563)	**<0.001**
Obese	42,234	250		
Posterior subcapsular cataract				
Bariatric surgery	42,329	62	0.397 (0.296, 0.533)	**<0.001**
Obese	42,311	156		
Diabetic cataract				
Bariatric surgery	41,999	173	0.326 (0.274, 0.386)	**<0.001**
Obese	42,129	533		
Cataract surgery				
Bariatric surgery	42,228	239	0.524 (0.448, 0.612)	**<0.001**
Obese	42,196	456		

Statistically significant values are in bold.

Concerning low vision and blindness, patients undergoing bariatric surgery were at significantly reduced likelihood of development compared to those who did not receive surgery (RR: 0.328; 95% CI: 0.294–0.365). When stratified by patients with only low vision and only blindness, those who underwent bariatric surgery were still significantly less likely to experience low vision (RR: 0.337; 95% CI: 0.302–0.377) and blindness (RR: 0.355; 95% CI: 0.274–0.460) (Table 5).

**Table 5:** Risk of post-operative low vision or blindness.

Cohorts	Patients in cohort	Patients with outcome	Risk ratio (CI)	*P* value
Blindness and Low Vision				
Bariatric surgery	41,714	435	0.328 (0.294, 0.365)	**<0.001**
Obese	41,772	1329		
Low vision				
Bariatric surgery	41,810	410	0.337 (0.302, 0.377)	**<0.001**
Obese	41,827	1217		
Blindness				
Bariatric surgery	42,251	78	0.355 (0.274, 0.46)	**<0.001**
Obese	42,314	220		

Statistically significant values are in bold.

## Discussion

The present study examining associations between bariatric surgery and common ocular pathology across a large national database, suggests bariatric surgery and associated systemic improvements may serve a protective role in development of ocular morbidity and mortality. Patients whose morbid obesity was managed surgery were significantly less likely to experience diabetic retinopathy, vitreous hemorrhage, intravitreal injection, vitrectomy, ocular hypertension, glaucoma, glaucomatous surgeries, use of ocular pressure lowering medications, age-related macular degeneration, cataract formation, cataract surgery, low vision, and blindness compared to patients who were not treated surgically.

This study largely supports and adds clarity to what has been reported in the literature regarding patients undergoing bariatric surgery and ocular pathology. With respect to diabetic retinopathy, this study supports findings of Akerblom et al. who found bariatric surgery to reduce future development of diabetic retinopathy in 5321 Swedish patients with diabetes. However, Akerblom et al also reported no difference in diabetic retinopathy complications such as development of proliferative DR, need for PRP, or IVI, which the present study finds to be reduced, possibly due to larger sample sizes leading to an appropriately powered analysis [15]. The present study also examines additional complications of DR not examined by Akerblom et al. such as development VH, finding reduced frequency in those whose obesity was managed surgically. The present data runs contrary to analyses of the STAMPEDE trial, which found no difference in DR incidence comparing patients who underwent bariatric surgery to intensive medical management. While these differences may be due to larger sample size in the present study, it is also plausible these differences are due to variations in the comparator group. In the STAMPEDE trial, patients undergoing surgery were compared to those undergoing intensive medical management of systemic disease while the present study compared outcomes to real world morbidly obese patients who were not managed surgically. Therefore, it is plausible the control cohort in the present study may have reduced access to care, decreased long term follow up, were poor candidates for surgery, or other differences unable to be controlled for in the study design. However, if this were the driving factor, it would be expected for this population to have reduced ophthalmic visits and diagnoses relative to the surgical cohort, potentially diminishing true effect of the surgical intervention. The diabetic cohorts as a whole suggest that treatment with bariatric surgery may be associated with reduced future development of diabetic retinopathy in morbidly obese patients along with sight-threatening complications of DR such as development of VH, TRD, DME, and need for treatment with IVI, and PRP.

The present study is also one of the first to examine bariatric surgery and associations with development of glaucomatous disease. To date, a retrospective study of 22 bariatric surgery patients by Shimonov et al. showed that BMI reduction was associated with significant and continued decline in IOP beyond 1 year after surgery. Specifically it was found that average BMI decreased from 41.9  ±  7.3 to 25.5  ±  5.7 kg/m^2^ at 1-year follow-up from bariatric surgery, which corresponded to a mean IOP decrease of 21% [16]. Additionally, small clinical cohorts examined by Burgansky-Eliash et al. have also shown bariatric surgery to reduce intraocular pressure [10]. Mechanistically, obesity associated orbital adipose tissue may elevate episcleral venous pressure, decreasing aqueous humor outflow resulting in IOP elevation [17]. Strong associations also exist between glaucoma and OSA, as carrying a diagnosis of OSA significantly increases the likelihood of being diagnosed with glaucoma [18]. These associations are thought to be a result of increased hypoxia in OSA, leading to optic nerve hypoxic damage and glaucomatous phenotype. Supporting these associations, patients who elected surgery were found to have lower incidences of being diagnosed with glaucomatous diseases such as ocular hypertension, glaucoma suspect, glaucoma, primary open angle glaucoma, and glaucomatous optic atrophy. Interestingly patients who underwent bariatric surgery were found to have significantly decreased incidence of OSA, in line with the above proposed mechanistic links between the two. In terms of clinical outcomes, surgically managed patients were less likely to use pressure lowering drops or undergo glaucoma surgeries in the future, highlighting relevant metrics for future analyses of clinical trials. The present study did not investigate secondary glaucoma mechanisms, such as neovascular glaucoma, which may also be affected by the many metabolic syndromes seen in morbidly obese patients.

While obesity has been identified as a risk factor for likelihood of being diagnosed with AMD, bariatric surgery has not been explored with respect to future development of disease. Although literature that explores the effect of bariatric surgery on ocular anatomy exists, there is a paucity of literature that explores AMD and bariatric surgery specifically. A prospective controlled study by Ozcelik-Kose et al. showed that total choroidal area and the choroidal vascularity index (CVI) exhibited significant increases when comparing the values 6 months before surgery and 6 months after surgery [19]. Similarly, in a prospective study of 40 patients, ElShazly et al. found significant increases in macular thickness and macular vascular density of the deep capillary plexus when compared 3 months before and 3 months after bariatric surgery [20]. Taken together these early studies may suggest systemic effects of bariatric surgery may include improvement in retinal microvascular circulation. Supporting these early associations, patients who underwent bariatric surgery were significantly less likely to develop nonexudative and exudative AMD. To more thoroughly examine these associations and impact on clinical treatment course, larger cohorts of surgical patients with long term follow up would be necessary.

Our study supports findings of Burkard et al., showing bariatric surgery to be associated with decreased risk of cataract diagnoses. However, the present study further adds novelty with the utilization of a large cohort of US patients, while also examining type of cataract developed and future need for cataract surgery [14]. Obesity has been identified as a significant risk factor for cataract formation, thought to be mediated through increases in reactive oxygen species and high circulating leptin in these patients, clouding the lens over time [21]. Diabetes is also a significant risk factor for cataract development, especially in those under 65 years of age, thought to be a result of increased osmotic and oxidative stress [22]. Because our cohorts are similar with respect to age and BMI at baseline, it is reasonable to assume that weight loss and systemic effects from bariatric surgery such as improved glycaemic control may have reduced likelihood of cataract development in our cohorts [4]. Furthermore, our findings show a reduction in incidence of all types of cataract including nuclear sclerotic, cortical, posterior subcapsular, and diabetic which have all been associated with obesity or diabetes [5, 22].

Supporting reductions seen in various ocular pathologies, patients in the bariatric cohort were found to be at significantly decreased risk of experiencing low vision or blindness post operatively. This finding largely aligns with data reported in the present study, as patients with bariatric surgery were less likely to develop DR, glaucomatous pathology, and AMD, all of which in isolation may contribute to low vision or blindness. While low vision and blindness is multifactorial in etiology and not all causes are investigated in the present study design, this finding is imperative to highlight that bariatric surgery not only results in reduced disease burden, but also reduces in visual decline in the process.

The findings of the present study, that morbidly obese patients who undergo bariatric surgery experience decreases in ocular morbidity and mortality, may be largely explained by improvements in systemic health as a result of the surgical intervention. Bariatric surgery has been shown long term to reduce BMI, improve glycaemic control, and reduce cardiovascular disease risk factors. Since DR is a microvascular complication of poor systemic diabetic health, it is reasonable to assume that the reduction in DR incidence may be explained by overall glycaemic improvement in this cohort. Reductions in incidence of glaucoma may be attributed to decreases in IOP or OSA over time in hand resulting in less disease development. Although the mechanistic link between obesity and AMD remains less clear, it is plausible to assume reductions in obesity, a studied risk factor for patients with AMD, is contributing to decreases in disease incidence observed. Future long term high powered clinical studies examining bariatric surgery and development of ocular pathology may add credence to the associations presented.

Strengths of the present study lie in the utilization of a national database to investigate bariatric surgery and its effect on the development of DR, glaucoma, AMD, and low vision and blindness in the future. To date, this is the largest longitudinal cohort to date examining these associations, allowing for analysis of a variety of pertinent ocular outcomes with respect to the above disease states. However, the present study is not without its limitations. Length of follow up is unable to be ascertained from the present data set, making it difficult to associate decreases in disease state seen with set chronologic time points. Even after utilization of propensity score matching, small baseline differences still existed in the two populations. However, these small differences may have been significant due to the large cohort sizes used, overpowering the measure of association. Because the data obtained through TriNetX is presented in aggregated form, it is difficult to assess patient-level data such as individual visual acuity, disease progression, and treatment course. While the present study reports univariate analysis as allowed by the TrinetX platform, subsequent analyses with incorporation of multivariate analysis would be of interest to further explore these associations. Additionally, it is also possible that patients receiving surgery may have been of higher socioeconomic status than those who did not pursue surgery. Unfortunately, this level of patient data was unable to be extracted as only ICD10 associated data was pulled. Finally, as TriNetX relies on ICD-10 and procedural codes to categorize variables, the conclusions drawn from such data is contingent on proper physician coding, leaving room for bias if disease states are coded inconsistently across health care organizations.

This study provides novel insight examining bariatric surgery and ocular pathology across a large national cohort in the US, uncovering a protective association between the surgery and ocular disease. The associations herein suggest a need for further investigation of bariatric surgery and obesity reduction on development of ocular morbidity and mortality in large long term clinical settings.

## Summary

### What was known before

- Bariatric surgery have been studied in association with ocular pathology, showing mixed but promising results.
- However, these studies do not often report postoperative disease development and do not investigate multiple disease pathologies.

### What this study adds

- This study is the first to examine the effect of bariatric surgery on risk of future ocular disease development across a large population, showing evidence that bariatric surgery may reduce development of multiple ocular diseases.

## Supplementary information

Supplemental table 1:

## Data Availability

The data that support the findings of this study are available from the authors but restrictions apply to the availability of these data, which were requisitioned from the TriNetX network, and so are not publicly available. Data are, however, available from the authors upon reasonable request.
