# Supplementary material for: Incidence of ocular pathology following bariatric surgery for with morbid obesity across a large United States National Database
Source: Eye (Lond). 2024 Apr 27;38(13):2603–9. doi: 10.1038/s41433-024-03088-z (PMC11385951; doi:10.1038/s41433-024-03088-z)
Supplement: Supplementary file 1 — Supplemental table 1: [file 41433_2024_3088_MOESM1_ESM.pdf]

Supplemental table 1: diagnoses and procedural codes used for outcomes

Outcome	ICD or procedural codes
**All forms of diabetic retinopathy**	E10.3, E11.3
**Nonproliferative diabetic retinopathy**	E10.32, E10.33, E10.43, E11.32, E11.33, E11.34
**Mild NPDR**	E10.32, E11.32
**Moderate NPDR**	E10.33, E11.33
**Severe NPDR**	E10.34, E11.34
**Proliferative diabetic retinopathy**	E10.35, E11.35
**Vitreous Hemorrhage**	H43.1
**Intravitreal injection**	67028, 1232150, 595060, 253337
**Pars Plana Vitrectomy**	67036, 1014238
**Pan Retinal Photocoagulation**	67228
**Tractional Retinal Detachment**	H33.4
**Diabetic macular edema**	E11.311, E10.311
**Retinal vascular occlusion**	H34
**Retinal vein occlusion**	H34.81, H34.83
**Retinal artery occlusion**	H34.1, H34.23
**Ocular hypertension**	H40.05
**Glaucoma suspect**	H40.0
**Glaucoma**	H40
**Primary Open Angle Glaucoma**	H40.11
**Glaucomatous optic atrophy**	H47.23

**Mild glaucoma**	H40.1231, H40.1131, H40.1111, H40.1121, H40.1191, H40.1211, H40.1221, H40.1291, H40.10X1
**Moderate glaucoma**	H40.10X2, H40.1232, H40.1332, H40.1132, H40.1122, H40.1112, H40.1192, H40.1222, H40.1212, H40.1292
**Severe glaucoma**	H40.10X3,H40.1233, H40.1133, H40.1123, H40.1113, H40.1193, H40.1223, H40.1213, H40.1293
**Glaucomatous surgeries and procedures**	Z98.83, 66761, 66170, 66172
**Use of pressure lowering eye drops**	43611, 283809, 1244607, 283810, 1992864, 8328,1988390, 14845, 134615, 1520, 10600, 60207, 194881
**Obstructive sleep apnea**	G47.33
**All forms of AMD**	H35.32, 35.31
**Non exudative AMD**	H35.31
**Exudative AMD**	H35.32
**Age related cataract**	H25
**Nuclear sclerotic cataract**	H25.1
**Posterior subcapsular cataract**	H25.04
**Cortical cataract**	H25.01
**Diabetic cataract**	E10.36, E11.36

**Cataract surgery**	110473004, 66830, 66840, 66850, 66852, 66920, 66940, 66983, 66984, 66987, 66988, 66984, 415089008, 1035657, 1035656, 66982, 66850, 361191005, 08DK3ZZ, 08DJ3ZZ
**Blindness and Low Vision**	H54
**Low vision**	H54.2, H54.3, H54.5, H54.6, H54.7
**Blindness**	H54.4, H54.0, H54.1, H54.8

Abbreviations: ICD = international classification of diseases, AMD= age related macular degeneration, NPDR = nonproliferative diabetic retinopathy,
